# *SlERF-RD1* acts as a developmental coordinator integrating plant canopy architecture and the ethylene-mediated ripening cascade in tomato

**DOI:** 10.1007/s00299-026-03848-z

**Published:** 2026-05-17

**Authors:** Sılanur Aydoğdu, Bayram Ali Yerlikaya, Seher Yerlikaya, Abdullah Aydın, Musa Kavas

**Affiliations:** Department of Agricultural Biotechnology, Faculty of Agriculture, Ondokuz Mayıs University, 55270 Samsun, Turkey

**Keywords:** Tomato, AP2/ERF, *SlERF-RD1*, Fruit ripening, Ethylene, Firmness, Cell wall, Plant architecture

## Abstract

**Key message:**

***SlERF-RD1*** **acts as a molecular brake, resulting in a decoupled ripening phenotype with delayed softening, while simultaneously optimizing plant architecture and flowering time, suggesting its potential for improving tomato shelf-life and productivity.**

**Abstract:**

Fruit ripening in tomato (*Solanum lycopersicum*) is a complex developmental process coordinated by a hierarchical network of transcription factors and the phytohormone ethylene. In this study, we identified and characterized *SlERF-RD1* (*Solyc02g077790*), a member of the AP2/ERF superfamily, as a strategic negative regulator of ripening and a coordinator of plant architecture. Transcriptomic meta-analysis revealed that *SlERF-RD1* is highly sensitive to ethylene and displays spatiotemporal enrichment in locular tissues, suggesting an early role in the ripening cascade. Stable overexpression of *SlERF-RD1* in tomato resulted in a significantly delayed onset of ripening, characterized by a 40–47% reduction in climacteric ethylene production and altered, tissue-specific carotenoid accumulation. Molecular analysis showed that these phenotypes are driven by the transcriptional down-regulation of the master regulator *SlRIN* and the rate-limiting carotenoid gene *SlPSY1*. Notably, *SlERF*-*RD1*-OE fruits maintained superior firmness during late ripening stages, which was correlated with the significant suppression of the cell wall-modifying genes *SlPG2A* and *SlEXP1*. Furthermore, we identified *Solyc01g108880* as a novel co-expressed target that is up-regulated explicitly at the red-ripe stage, suggesting a late-stage cell wall reinforcement mechanism. Beyond fruit attributes, *SlERF-RD1* overexpression reconfigured plant architecture into a more compact canopy and accelerated the floral transition by up-regulating *SlSFT*. Our findings demonstrate that *SlERF-RD1* promotes an asynchronous, firm-ripe fruit phenotype, while supporting optimized vegetative growth. This study highlights *SlERF*-*RD1* as a high-potential target for genetic strategies aimed at enhancing both crop architecture and post-harvest shelf-life.

**Supplementary Information:**

The online version contains supplementary material available at 10.1007/s00299-026-03848-z.

## Introduction

Tomato (*Solanum lycopersicum* L.) is the primary model for climacteric fruit ripening, a developmental shift governed by a substantial increase in autocatalytic ethylene production, known as *System II ethylene synthesis* (Alba et al. 2005). This transition is initiated by the transcriptional activation of key biosynthetic genes, primarily *SlACS2*, *SlACS4*, and *SlACO1*, which elevate ethylene levels to trigger a cascade of ripening-related physiological changes (Giovannoni et al. 2017). The ethylene signal is detected by membrane-bound receptors (e.g., SlETR1–7) and transmitted via a conserved pathway in which *SlCTR1* acts as a negative regulator and *SlEIN2* functions as a positive transducer, resulting in the stabilization of EIN3/EIL transcription factors in the nucleus (Mata et al. 2018).

As terminal components of the ethylene signaling pathway, members of the *APETALA2*/*Ethylene Response Factor* (*AP2*/*ERF*) superfamily act as downstream effectors that convert hormonal signals into specific ripening outputs. Recent studies have highlighted the role of ERFs in modulating the expression of "master regulators" such as *MADS*-*RIN*, *NAC*-*NOR*, and *CNR*, creating a complex feedback loop that fine-tunes the ripening rate (Deng et al. 2022). While many ERFs promote ripening, the identification of negative regulators, such as SlERF.F12 and SlLBD33, has revealed an essential "molecular braking" mechanism that prevents precocious maturation and ensures proper nutrient partitioning (Liu et al.^2020^; Deng et al. 2022). The initiation and progression of tomato fruit ripening require a highly coordinated interplay between climacteric ethylene production and an evolving hierarchy of master transcription factors, prominently, including *MADS-RIN, NAC-NOR,* and *NOR-like1* (Huang et al. 2025; Aprilyanto et al. 2025).

One of the most critical outputs of this regulatory network is the modification of the primary cell wall and middle lamella. The coordinated action of enzymes such as polygalacturonases (PGs), pectin methylesterases (PMEs), and expansins (EXPs) leads to the progressive disassembly of the pectin-cellulose-hemicellulose network, resulting in fruit softening (Smith et al. 1988; Brummell et al. 1999). Although softening is essential for palatability, excessive cell wall degradation significantly limits post-harvest shelf-life and increases susceptibility to opportunistic pathogens. While numerous regulators of ripening have been characterized, the exact transcriptional mechanisms that separate softening from other ripening traits and thus generate “firm-ripe” phenotypes remain largely unclear.

Recent discoveries continue to expand the multifaceted roles of AP2/ERF transcription factors within this ripening network; for instance, the newly characterized SlERF.D6 has been demonstrated to positively regulate ripening by orchestrating downstream transcriptional cascades in both pericarp and locule tissues (Chen et al. 2025a). In this study, we characterize *SlERF-RD1 (Solyc02g077790)*, a distinct member of the AP2/ERF family identified through high-resolution transcriptomic data mining. Unlike many ripening-induced factors, *SlERF*-*RD1* displays a distinct spatiotemporal expression pattern with a strong affinity for locular tissues and a high sensitivity to ethylene-related perturbations. By utilizing a stable overexpression approach in tomato cv. Crocker, we provide molecular evidence that *SlERF*-*RD1* functions as a strategic repressor of the ripening cascade. We show that it significantly reduces the climacteric ethylene peak by modulating upstream regulators and enhances fruit structural integrity through the targeted suppression of *SlPG2A* and *SlEXP1*. Furthermore, the pleiotropic influence of *SlERF*-*RD1* on plant canopy architecture and flowering time suggests a multifaceted role in coordinating the transition from vegetative to reproductive growth, offering a novel genetic target for optimizing both crop yield and post-harvest longevity.

## Materials and methods

### In silico analysis and candidate gene identification

To identify high-priority ripening regulators within the AP2/ERF superfamily, transcriptomic meta-analyses were conducted using the Genevestigator database (Hruz et al. 2008). Genevestigator has been widely used as a transcriptomic meta-analysis platform for integrating publicly available expression datasets across multiple plant species (Zimmermann et al. 2004; Hruz et al. 2008). Although the platform is no longer actively maintained, it remains a valuable resource for mining historically curated datasets generated from independent studies. In this study, Genevestigator was employed as an initial screening tool to identify candidate genes based on consistent expression patterns across developmental stages and experimental perturbations. To ensure robustness and reproducibility, all expression trends identified through Genevestigator were cross-validated using independent datasets from the Tomato Expression Atlas (Fernandez-Pozo et al. 2017). The transcriptomic data were retrieved from the Genevestigator platform (Hruz et al. 2008), using the experiment SL-00038, which integrates publicly available tomato fruit developmental RNA-seq datasets. The perturbations shown correspond to stage-based comparisons (e.g., 1 cm, 2–3 cm, mature green, breaker, and red-ripe) generated within the Genevestigator analytical framework. These data are derived from multiple publicly available transcriptomic studies, including resources, such as the Tomato Expression Atlas (Fernandez-Pozo et al. 2017), and are visualized using the 3D Expression Cube. For functional categorization, Gene Ontology (GO) and KEGG pathway enrichment analyses were performed on the identified co-expression modules using the clusterProfiler R package (Wu et al. 2021) with a significance threshold of FDR < 0.05. Co-expression relationships were inferred based on pairwise Pearson correlation analysis of gene expression profiles across developmental stages within the Genevestigator dataset. Genes exhibiting high correlation coefficients (*r* ≥ 0.85) with *SlERF-RD1* were selected to construct the co-expression module. The resulting gene set was visualized using hierarchical clustering (Euclidean distance, complete linkage) implemented in R to generate the heatmap representation. To investigate the potential molecular interactions between *SlERF-RD1* and its target genes, the 2000 bp upstream regions of *SlRIN* (Solyc05g012020), *SlEXP1* (Solyc06g051800), *SlPSY1* (Solyc03g031860), and *SlPG2A* (Solyc10g080210), along with the *SlERF-RD1* (Solyc02g077790) promoter itself, were retrieved from the Phytozome v14. These sequences were systematically scanned for cis-acting regulatory elements using the PLACE (Plant Cis-acting Regulatory DNA Elements) database. The search specifically targeted high-confidence binding motifs for AP2/ERF family members, including GCC-boxes (AGCCGCC; AGCBOXNPGLB) and W-boxes (TGACY/TTGAC; WBOXNTERF3 and WBOXATNPR1). Additionally, the analysis included the identification of dehydration-responsive elements (DRE/CRT; RCCGAC), ethylene-responsive elements (ERELEE4; AWTTCAAA), and developmental regulators such as DOF-core motifs (AAAG; DOFCOREZM). The spatial distribution and exact genomic coordinates of these elements relative to the transcription start site (TSS) were mapped to provide a theoretical framework for the observed transcriptional repression in *SlERF-RD1*-OE lines.

### Plant materials and growth conditions

Tomato (*Solanum lycopersicum* L. cv. Crocker) was used as the biological material for all experiments, including genetic transformation and physiological analyses. Seeds were surface-sterilized and germinated on half-strength Murashige and Skoog (MS) medium. Seedlings were then transplanted into organic soil and grown in a climate-controlled greenhouse at the Department of Agricultural Biotechnology, Ondokuz Mayıs University. The growth conditions were maintained at a 25 °C day/20 °C night temperature cycle with a 16 h light/8 h dark photoperiod and a relative humidity of 60%. Plant cultivation, irrigation, and fertilization were performed according to the protocols established in our previous studies (Gökdemir et al. 2022; Aydin et al. 2025). Fruit samples were harvested at three distinct developmental stages: Mature Green (MG, ~ 35 days post-anthesis), Breaker (BR, onset of color change), and Red-Ripe (RR, 7 days after the breaker stage), and immediately processed or flash-frozen in liquid nitrogen for downstream molecular analyses (Giovannoni 2004).

### Vector construction and tomato transformation

The full-length coding sequence (CDS) of *Solyc02g077790* (*SlERF-RD1*) was amplified via RT-PCR using total RNA extracted from *S. lycopersicum* cv. Crocker. RNA isolation was performed with the RNeasy Plant Mini Kit (Qiagen), followed by an on-column DNase I treatment to eliminate genomic DNA contamination. cDNA was synthesized from total RNA using the iScript™ cDNA Synthesis Kit (Bio-Rad) according to the manufacturer’s instructions.

To ensure directional and seamless cloning, *SlERF-RD1*-specific primers were designed using the In-Fusion® primer design tool (Takara Bio), incorporating 15-bp overlapping sequences homologous to the insertion site of the binary vector. The binary expression vector pIKB004, which drives transgene expression under the control of the constitutive CaMV 35S promoter, was linearized by double digestion with *SalI* and *HindIII* restriction enzymes (New England Biolabs). The purified PCR product and the linearized vector were assembled using the In-Fusion® HD Cloning Kit (Takara Bio).

The resulting recombinant construct was transformed into *Escherichia coli* DH5α competent cells using the heat-shock method and selected on LB agar supplemented with spectinomycin (100 mg/L). Positive clones were initially identified through colony PCR using 35S forward and NOS reverse primers and further validated by Sanger sequencing to ensure sequence integrity. The confirmed plasmid was subsequently introduced into *Agrobacterium tumefaciens* strain GV3101 by electroporation. Transformants were selected on LB medium containing spectinomycin (100 μg/L), gentamicin (50 μg/L), and rifampicin (25 μg/L).

### *Agrobacterium*-mediated transformation and generation of transgenic lines

Stable genetic transformation of tomato (cv. Crocker) was performed using *Agrobacterium tumefaciens* strain GV3101 harboring the pIKB004:*SlERF-RD1*(Fig. S1a) construct. Transformation was performed using the cotyledon explant method, as previously described in our established protocols (Gökdemir et al. 2022; Aydin et al. 2025).

Briefly, cotyledon segments from 8-day-old in vitro germinated seedlings were pre-cultured and subsequently co-cultivated with the *Agrobacterium* suspension for 48 h in the dark. Explants were then transferred to a selective regeneration medium containing hygromycin (for plant selection) and cefotaxime (to inhibit *the growth of Agrobacterium*). Regenerated shoots were excised and transferred to a rooting medium. Putative T₀ transgenic plants were initially screened by genomic PCR using vector-specific primers (35S-F and *SlERF-RD1* -R) to confirm the integration of the T-DNA cassette (Table S1; Fig.S1b). The positive T₀ lines were grown to maturity in the greenhouse to obtain T₁ seeds. Homozygous T₂ lines (#*4*, #*5*, and #*7*) were identified through segregation analysis and confirmed by qRT-PCR for overexpression of the target gene (Fig.S1c) before being used for further phenotypic and physiological characterization.

### Phenotypic and morphological characterization

Vegetative and reproductive growth parameters were systematically evaluated to assess the pleiotropic effects of *SlERF-RD1* overexpression. Plant height (from the soil surface to the apical meristem) and canopy diameter were measured 30 days after germination (DAG). To quantify canopy architecture, zenithal photographs of the plants were captured and analyzed for surface area coverage using ImageJ software (National Institutes of Health, USA) as described by Abràmoff et al. (2004).

Reproductive characterization included the determination of the floral transition period (days from germination to the first anthesis) and the total number of flowers per inflorescence for the first three clusters. Floral organ morphology, specifically the total area (mm^2^) of petals and sepals, was quantified using a high-resolution flatbed scanner followed by image processing in ImageJ. These morphological assessments were conducted in accordance with the phenotypic descriptors for tomato established by Shinozaki et al. (2018) and our previous studies (Gökdemir et al. 2022; Aydin et al. 2025).

### Physiological fruit analysis: firmness, color, and ethylene production

To characterize the ripening problems in *SlERF-RD1*-OE lines, physiological parameters were measured at MG, BR, and RR stages. Fruit firmness was quantified using a digital penetrometer (Lutron FG-5020) equipped with a 5-mm diameter plunger. Measurements were taken at three equidistant points along the equatorial plane of each fruit, and the results were expressed in Newtons (N) as described by Liu et al. (2020).

External fruit color was evaluated using a Minolta Chromameter (CR-400, Konica Minolta, Japan). The color was recorded using the CIELAB scale, where L* represents lightness, a* represents redness/greenness, and b* represents yellowness/blueness. The chroma (C*) and hue angle (*h*^0^) were calculated according to McGuire (1992).

Ethylene production was determined by placing individual fruits of known weight in airtight 1-L glass jars for 3 h at 25 °C. A 1-mL gas sample was withdrawn from the headspace using a syringe and injected into a gas chromatograph (Shimadzu GC–MS-QP2010S) equipped with a flame ionization detector (FID) and a capillary column. Ethylene concentration was calculated against a standard curve and expressed as *μL.kg*^*−1*^*.h*^*−1*^ following the methodology of Alba et al. (2005).

### Determination of soluble solids and vitamin C content

The internal biochemical quality of the fruits was assessed at the RR stage. Soluble solids content (SSC), expressed as °Brix, was measured using a digital refractometer (Atago PAL-1, Tokyo, Japan). Representative fruit samples were homogenized, and the resulting juice was filtered before being placed on the refractometer prism at a constant temperature of 25 °C, following the method described by Beckles (2012).

Vitamin C (ascorbic acid) concentration was determined using the 2,6-dichlorophenolindophenol (DCPIP) titrimetric method. Briefly, 5 g of fruit tissue was homogenized in a 5% (w/v) metaphosphoric acid solution to prevent ascorbic acid oxidation. The extract was centrifuged, and the supernatant was titrated with a standardized DCPIP solution until a persistent pink color was observed. The results were calculated as mg of ascorbic acid per 100 g of fresh weight (FW) according to Stevens et al. (2007).

### RNA extraction and quantitative real-time PCR (qRT-PCR)

Total RNA was extracted from various tomato tissues, including vegetative organs and fruit samples, at MG, BR, and RR stages, using the RNeasy Plant Mini Kit (Qiagen, Hilden, Germany) according to the manufacturer’s instructions. To prevent genomic DNA contamination, an on-column DNase I (Qiagen) treatment was integrated into the extraction protocol. The purity and concentration of the RNA were assessed using a NanoDrop™ One spectrophotometer (Thermo Fisher Scientific, USA), and RNA integrity was verified via agarose gel electrophoresis.

First-strand cDNA synthesis was performed from 1 μg of total RNA using the iScript™ cDNA Synthesis Kit (Bio-Rad, Hercules, CA, USA). qRT-PCR was conducted on a Bio-Rad CFX96 Real-Time PCR Detection System using the SsoAdvanced™ Universal SYBR® Green Supermix (Bio-Rad). The thermal cycling conditions and reaction setup were adjusted according to the manufacturer’s recommendations. Relative transcript abundance was calculated using the 2^−ΔΔCT^ method as described by Livak and Schmittgen (2001). The tomato *SlActin* (*Solyc03g078400*) gene was utilized as an internal constitutive reference for normalization (Expósito-Rodríguez et al. 2008). All oligonucleotide primers used for cloning and qRT-PCR analysis in this study are listed in Table S1. All experiments were performed with three biological and three technical replicates.

### Statistical analysis

All experiments were conducted using a completely randomized design with at least three biological replicates. To evaluate the effect of *SlERF-RD1* overexpression, statistical significance between the WT and each independent transgenic line (OE) was determined using Student’s t-test. For analyses involving multiple ripening stages or tissue types, data were subjected to a one-way analysis of variance (ANOVA) followed by post-hoc comparisons.

Differences were considered statistically significant at *P* < 0.05 (*), *P* < 0.01 (**), or *P* < 0.001 (***). Pearson correlation coefficients (r) were calculated to assess the transcriptional synergy between *SlERF-RD1* and its co-expressed partners. All statistical computations and high-resolution data visualizations, including bar plots and heatmaps, were generated using GraphPad Prism (Version 9.0) and R software (R Core Team 2021).

## Result

### *SlERF-RD1* as a pleiotropic developmental coordinator: multi-layered bioinformatic prioritization and spatiotemporal characterization

To identify primary transcriptional nodes that govern tomato fruit maturation, we implemented an integrated discovery pipeline that leveraged large-scale transcriptomic datasets. This systematic screening highlighted *SlERF-RD1*, a member of the AP2/ERF superfamily, as a high-confidence candidate due to its robust association with ripening-inductive signals.

**Fig. 1:**
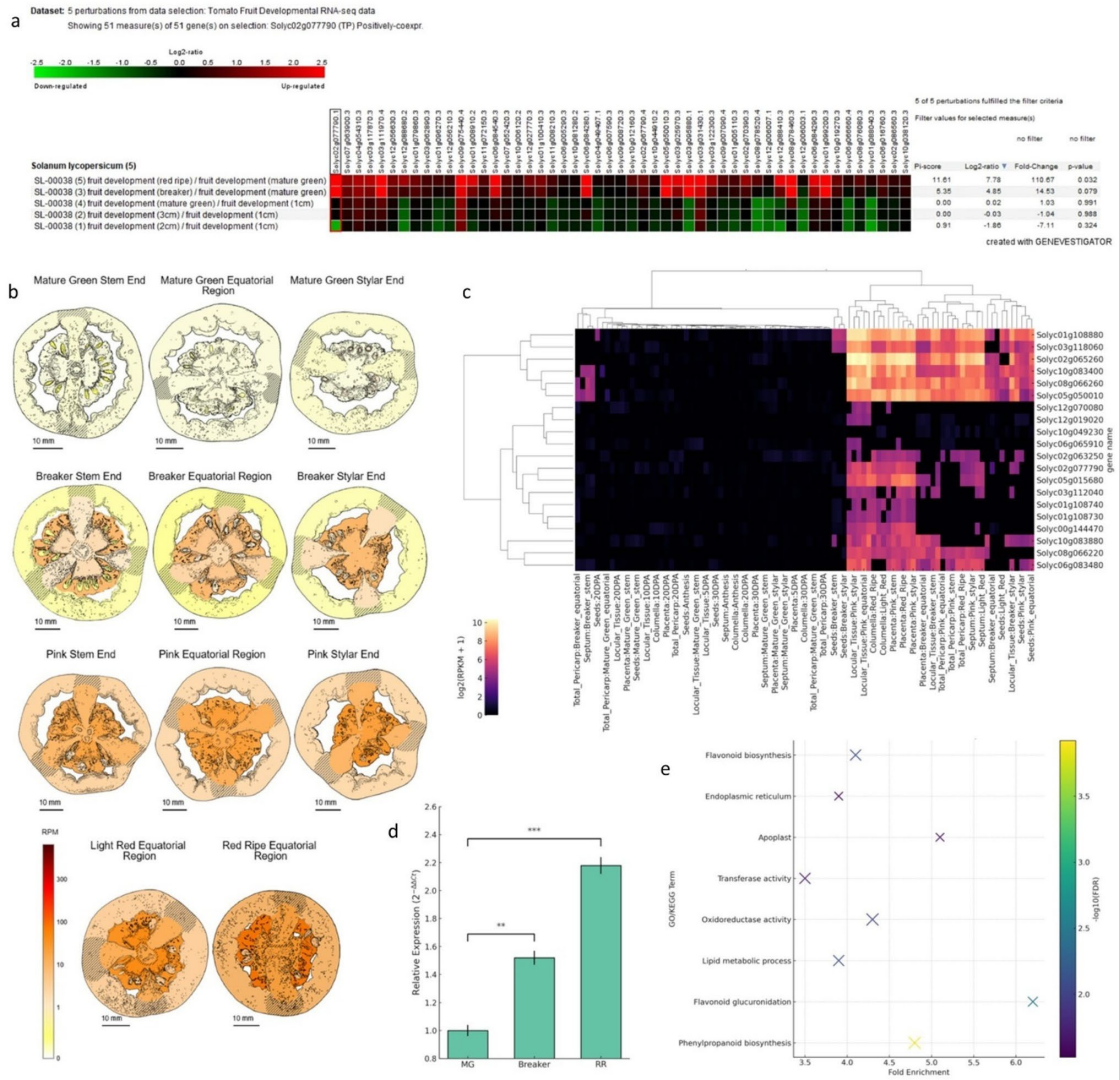
Integrated discovery and spatiotemporal characterization of *SlERF-RD1*. **a** Heatmap illustrating the transcriptional response of *SlERF-RD1* across five developmental perturbations derived from the Genevestigator experiment SL-00038, representing stage-specific comparisons during tomato fruit development. **b** High-resolution visualization of *SlERF-RD1* transcript distribution within the tomato fruit across Mature Green (MG), Breaker (BR), and Red-Ripe (RR) stages, highlighting tissue-specific enrichment in the pericarp, septum, and locular tissues. **c** Co-expression Heatmap: Identification of a synchronized ripening-associated gene module. The heatmap displays the top-ranked genes exhibiting high transcriptional plasticity and synergy with *SlERF-RD1*, including the cell-wall-related candidate *Solyc01g108880*. **d** Empirical validation of ripening-inductive expression: qRT-PCR analysis of *SlERF-RD1* transcript abundance at MG, BR, and RR stages. Relative expression levels were normalized to *SlActin*, and the MG stage was utilized as the calibrator to calculate fold-changes across ripening stages. **e** Functional enrichment analysis: GO and KEGG pathway enrichment of the *SlERF-RD1* co-expression module, identifying dominant associations with Phenylpropanoid biosynthesis and cell wall modification (FDR = 1.2e-4). Data in (D) represent the mean ± SD of three biological replicates. Asterisks denote significant differences based on Student’s t-test (**P < 0.01; ***P < 0.001)

Detailed analysis of transcriptomic changes using the Genevestigator platform revealed that *SlERF-RD1* expression is highly sensitive to the ripening-triggering machinery (Fig. 1a). Analysis of the Genevestigator SL-00038 dataset across five distinct fruit developmental stages (from 1 cm to red-ripe) revealed consistent up-regulation of *SlERF-RD1*, corroborating its placement within the core maturation network. To refine the spatiotemporal activity of *SlERF-RD1*, we utilized high-resolution data from the Tomato Expression Atlas (TEA) (Fig. 1b; Fig. S2). The expression landscape, visualized through a 3D Expression Cube, revealed a profound fruit-specific induction, with transcripts being predominantly localized in the pericarp, septum, and locular tissues during the transition from Mature Green (MG) to the Red-Ripe (RR) stage (Fig. 1b; Fig. S2a and S2b). Notably, the expression intensity in the locule reached its peak at the RR stage, exhibiting a strong transcriptional correlation with placental tissues (Fig. 1b; Fig. S2c; Pearson *r* = 0.91).

A pivotal finding in our discovery phase was the identification of a highly synchronized co-expression gene module associated with *SlERF-RD1*. The heatmap analysis revealed a cluster of genes that exhibit nearly identical transcriptional patterns across developmental stages and physiological disorders (Fig. 1c). This module was defined based on high Pearson correlation coefficients (*r* ≥ 0.85) across developmental stages, indicating strong transcriptional co-regulation rather than direct functional interaction. Among the most tightly correlated partners, we identified Solyc01g108880, a co-expressed candidate gene whose expression profile suggests a potential association with cell wall-related processes and secondary metabolism. The high transcriptional synergy within this module suggests that *SlERF-RD1* acts as a central hub, orchestrating the expression of downstream effector genes required for the ripening transition. The in silico expression pattern was empirically validated via qRT-PCR across three canonical ripening stages. Consistent with our computational predictions, the relative expression of *SlERF-RD1* exhibited a progressive up-regulation as ripening advanced. Specifically, using the MG stage as a baseline (assigned a value of 1), *SlERF-RD1* transcript levels showed a significant ~ 1.5-fold increase at the Breaker (BR) stage, subsequently reaching a 2.1-fold peak at the RR stage (Fig. 1d; *P* < 0.01). Although the magnitude of induction is moderate, the steady increase from MG to RR reinforces the role of *SlERF-RD1* as a constitutive component of the ripening-associated transcriptional program. To delineate the functional implications of this ripening-associated induction, we performed GO and KEGG enrichment analyses on the *SlERF-RD1* co-expression module. The analysis revealed a highly specific overrepresentation of genes involved in phenylpropanoid biosynthesis and cell wall modification, particularly those targeting hemicellulose and pectin metabolic pathways (Fig. 1e; FDR = 1.2e-4). Collectively, these multi-layered bioinformatic and empirical hallmarks establish *SlERF-RD1* as a strategic regulatory node orchestrating tomato fruit ripening and structural integrity downstream of ethylene signaling. It is important to note that this association is based on co-expression analysis and does not imply a direct functional or regulatory relationship.

### Generation and molecular validation of *SlERF-RD1* overexpression lines

To investigate the functional role of *SlERF-RD1* in tomato fruit development and maturation, we developed stable transgenic lines overexpressing the full-length CDS of *SlERF-RD1* under the control of the constitutive CaMV35S promoter (Fig.S3a). Stable integration of the T-DNA was confirmed in five independent T0 transformants (lines #2, #4, #5, #6, and #7) via genomic PCR using vector-specific primers, which yielded the expected 634 bp amplicon (Fig.S3b). To assess the magnitude of overexpression, we quantified transcript abundance through qRT-PCR. Three independent lines (#4, #5, and #7) displayed substantial and statistically significant up-regulation of *SlERF-RD1*, with expression levels ranging from 4- to 11-fold relative to wild-type (WT) plants (Fig.S3c). Specifically, lines #5 and #7 exhibited the highest transcript accumulation (*P* < 0.001), while line #4 showed a robust but comparatively lower level of overexpression than those (*P* < 0.001). Based on these molecular profiles, these three lines were selected for further phenotypic and physiological characterization.

### *SlERF-RD1* reconfigures plant architecture and accelerates the floral transition

**Fig. 2:**
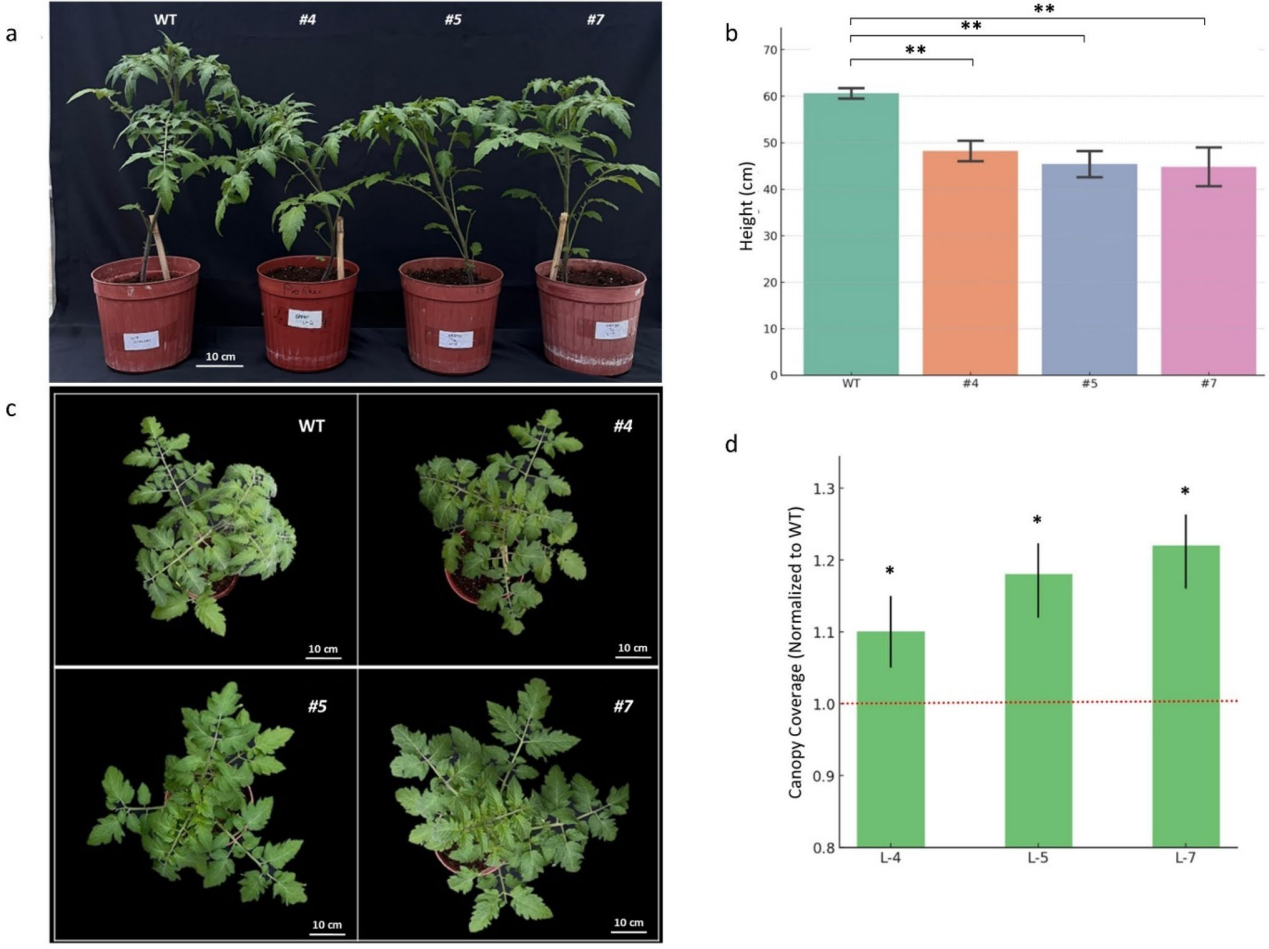
Overexpression of *SlERF-RD1* reconfigures tomato plant architecture. **a** Representative phenotypes of 30-day-old WT and *SlERF-RD1*-OE lines (#*4*, #*5*, and #*7*), showing a more compact growth habit in transgenic plants. **b** Quantitative analysis of plant height (cm), showing a significant reduction in vertical growth in OE lines. **c** Top-down view of canopy architecture, highlighting the increased lateral expansion in OE lines. **d** Normalized canopy coverage (%) calculated from image-based surface area analysis. Data represent mean ± SD (*n* = 10). Asterisks indicate statistical significance relative to WT based on Student’s t-test (**P < 0.01; ***P < 0.001)

To evaluate the broader developmental impact of *SlERF-RD1*, we first characterized the vegetative and reproductive growth patterns of the overexpression (OE) lines. Phenotypic monitoring at 30 days after germination (DAG) revealed that constitutive overexpression of *SlERF-RD1* significantly reconfigures tomato plant architecture, resulting in a more compact and robust growth habit (Fig. 2a). Quantitative analysis confirmed a 20–26% reduction in plant height in the OE lines compared to wild-type (WT) plants (*P* < 0.01; Fig. 2b). Notably, this reduction in vertical elongation was accompanied by a significant increase in lateral expansion; the projected canopy coverage of OE lines was 10–22% greater than that of the WT (Fig. 2c and d), indicating a strategic shift in biomass allocation toward lateral development.

**Fig. 3:**
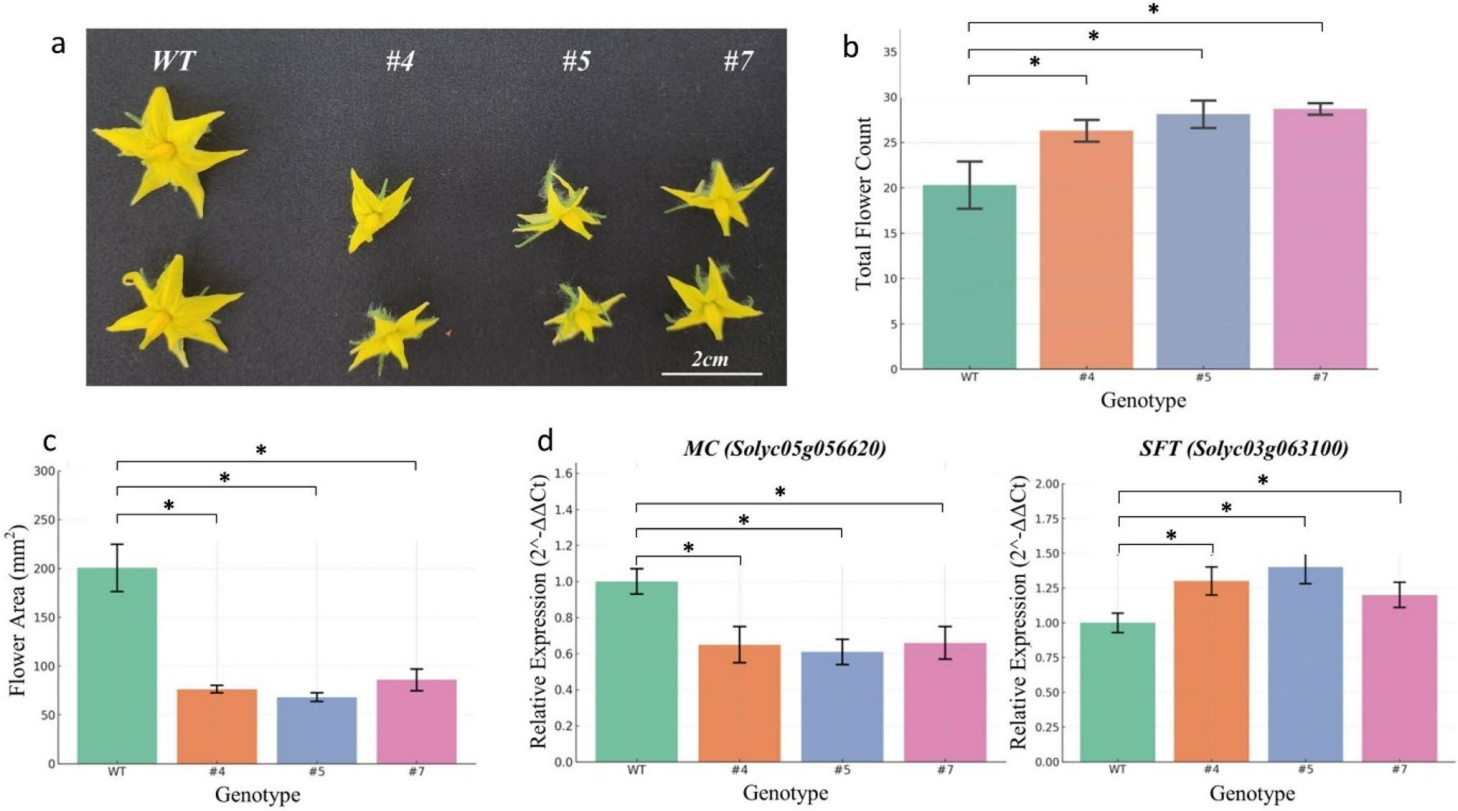
*SlERF-RD1* promotes early floral transition and increases reproductive productivity while restricting floral organ size. **a** Visual comparison of flower morphology and anthesis timing between WT and *SlERF-RD1*-OE lines (#*4*, #*5*, and #*7*), showing more compact floral architecture in transgenic plants. **b** Quantitative analysis of the total number of flowers produced on the first three inflorescences, highlighting the significantly increased reproductive output in OE lines. **c** Measurement of total floral area (mm^2^), revealing a significant reduction in flower size in *SlERF-RD1-*overexpressing lines. **d** Relative mRNA abundance of the florigen gene *SFT* and the calyx development regulator *MC* in apical meristems (14–18 DAG) determined by qRT-PCR. Data represent mean ± SD (*n* = 10 for phenotypic measurements; *n* = 3 biological replicates for gene expression analysis). Asterisks indicate statistical significance relative to WT based on Student’s t-test (*P < 0.05; **P < 0.01; ***P < 0.001)

Parallel to the architectural modifications observed, *SlERF-RD1*-OE lines exhibited a significantly accelerated transition to reproductive development, reaching anthesis 4–7 days earlier than the wild-type (WT) (*P* < 0.05; Fig. 3a). A marked enhancement in reproductive output accompanied this precocious flowering; the number of flowers on the first three inflorescences was substantially higher in the OE lines, averaging 26.3–28.7 flowers per cluster, compared to 20.2 ± 2.3 in the WT (*P* < 0.05; Fig. 3b). Interestingly, while the total flower count increased, the individual floral area was significantly reduced in the OE lines (WT: 195.7 ± 22.1 mm^2^ vs. OE: 66.2–86.3 mm^2^; *P* < 0.001; Fig. 3c), a trade-off visually manifested in the more compact floral morphology. To elucidate the transcriptional basis of this heterochronic shift, we quantified key regulatory markers in apical meristems (14–18 DAG). The florigen-encoding gene SINGLE FLOWER TRUSS (SFT) was significantly up-regulated in all OE lines (*P* < 0.05; Fig. 3d), whereas the calyx development regulator MACROCALYX (MC) was strongly suppressed, exhibiting up to a 39% reduction in transcript levels (*P* < 0.05; Fig. 3d). Collectively, these findings demonstrate that *SlERF-RD1* promotes early flowering and increased floral density while concurrently restricting organ expansion by modulating the SFT-MC regulatory axis.

### *SlERF-RD1* overexpression postpones ripening progression and modulates fruit quality attributes

**Fig. 4:**
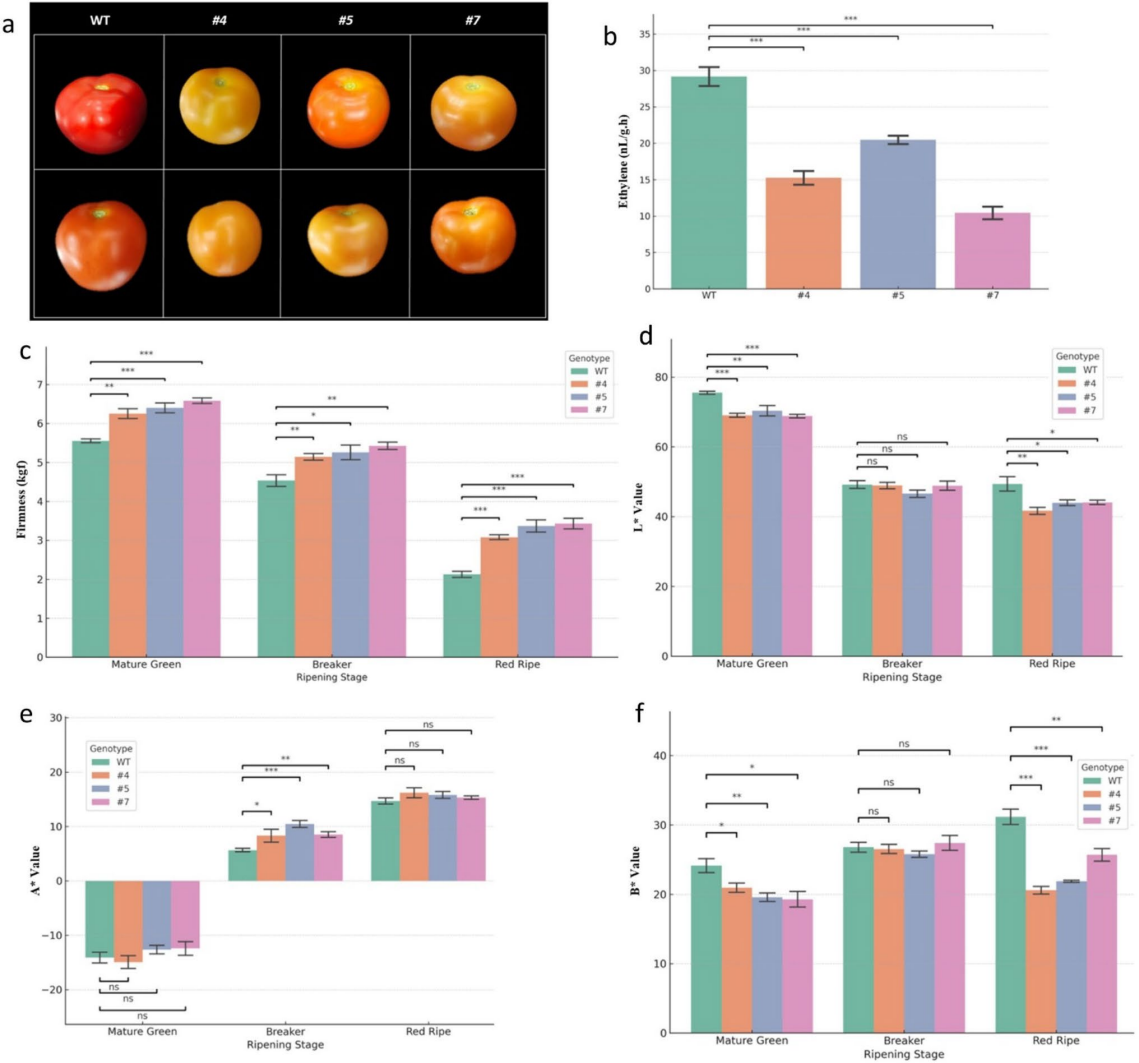
Physiological and colorimetric characterization of *SlERF-RD1*-OE fruits during ripening. **a** Representative phenotypes of WT and OE fruits showing delayed external pigmentation at the red-ripe stage. **b** Ethylene production rates (nL g^−1^ h^−1^) at the RR stage, showcasing significantly reduced biosynthesis in OE lines. **c** Firmness progression across mature green (MG), breaker (BR), and red-ripe (RR) stages. CIELAB color coordinates representing **d** L* (lightness), **e** a* (redness), and **f** b* (yellowness). Data represent mean ± SD. Asterisks indicate statistical significance (*P < 0.05; ** *P* < 0.01; ***P < 0.001, Student’s t-test)

To evaluate the impact of *SlERF-RD1* on the physiological hallmarks of tomato maturation, we performed a multi-parametric analysis of ripening dynamics. The most prominent effect of *SlERF-RD1* overexpression was a substantial delay in external color transition; while wild-type (WT) fruits reached a characteristic deep-red state at the Red-Ripe (RR) stage, all transgenic lines exhibited a persistent yellow–orange hue, indicating an asynchronous ripening program (Fig. 4a). Consistent with this phenotype, the climacteric ethylene burst was significantly suppressed in the overexpression lines. The RR stage, defined as 7 days post-breaker, was specifically evaluated to determine whether the repressive effects of *SlERF-RD1* were merely a transient delay of the climacteric peak or a sustained inhibition of the maturation program. At this time point, ethylene production in WT fruits reached 29.1 ± 1.3 nL g⁻^1^ h⁻^1^, whereas *SlERF-RD1*-OE lines exhibited a 40–47% reduction, with emission rates ranging from 15.3 ± 0.9 to 17.8 ± 1.1 nL g⁻^1^ h⁻^1^ (P < 0.001; Fig. 4b). This significant reduction in ethylene evolution likely serves as a primary driver for the overall deceleration of the maturation process. Furthermore, the maintenance of fruit structural integrity was significantly enhanced in the transgenic lines. Quantitative assessment of fruit firmness across ripening stages revealed that while WT fruits underwent rapid softening, *SlERF-RD1*-OE lines maintained markedly higher resistance to penetration (Fig. 4c). Even at the RR stage, OE fruits remained significantly firmer than the WT (*P* < 0.001), suggesting that *SlERF-RD1* may function as a negative regulator of the cell wall disassembly machinery. Pigmentation alterations were further quantified using CIELAB colorimetric parameters. For the L* parameter (Fig. 4d), no statistically significant difference was observed at the Breaker (BR) stage, as indicated by “ns” in the figure, whereas significant differences were clearly detected at both the Mature Green (MG) and Red-Ripe (RR) stages, consistent with the statistical analysis. Interestingly, while the macroscopic visual appearance of the OE fruits was distinctly pale/orange at the RR stage (Fig. 4a), the colorimetric data revealed a decoupling between visual phenotype and instrument readings. The a* value (green-to-red axis) in OE fruits remained slightly higher than or comparable to the WT (Fig. 4e). Conversely, the b* value (yellowness) was significantly lower in the transgenic lines (Fig. 4f). This apparent visual-colorimetric paradox is driven by a distinct spatial ripening delay in the OE lines. Sectioning of the fruits revealed that while internal tissues accumulated red pigments similarly to the WT, the external epidermis and outer pericarp remained distinctly pale (Fig. S4). The Minolta CR-400 chromameter’s intense xenon flash penetrates the shallow pale outer layers to detect the underlying internal red pigments, yielding the high a* reading. However, visual perception under ambient light predominantly captures surface reflectance from the pale epidermis, resulting in the perceived orange/pale phenotype. Furthermore, the significantly reduced b* value likely reflects an impaired accumulation of epidermal yellow flavonoids. Collectively, these data demonstrate that *SlERF-RD1* overexpression causes an asynchronous, tissue-specific delay in fruit pigmentation. While longitudinal storage trials were not conducted, the 40–47% reduction in ethylene emission and the significantly maintained fruit firmness observed up to 7 days post-breaker serve as potential physiological indicators of an enhanced shelf-life potential in *SlERF-RD1*-OE lines. Beyond these physiological changes, *SlERF-RD1* positively modulated internal fruit quality. Analysis of primary metabolites at the RR stage revealed a consistent increase in soluble solids content (°Brix), ranging from 5.2 to 5.8% in OE lines, compared to 4.6% in the WT. Notably, ascorbic acid (Vitamin C) levels were significantly elevated, with OE fruits accumulating up to 21.2 mg/100 g FW, representing an approximate 43% increase over the WT (*P* < 0.05). Collectively, these findings demonstrate that *SlERF-RD1* orchestrates a distinct ripening program that prioritizes shelf-life and nutritional density over rapid pigmentation.

### Transcriptional reprogramming of ripening-related pathways in* SlERF-RD1-*OE fruits

**Fig. 5:**
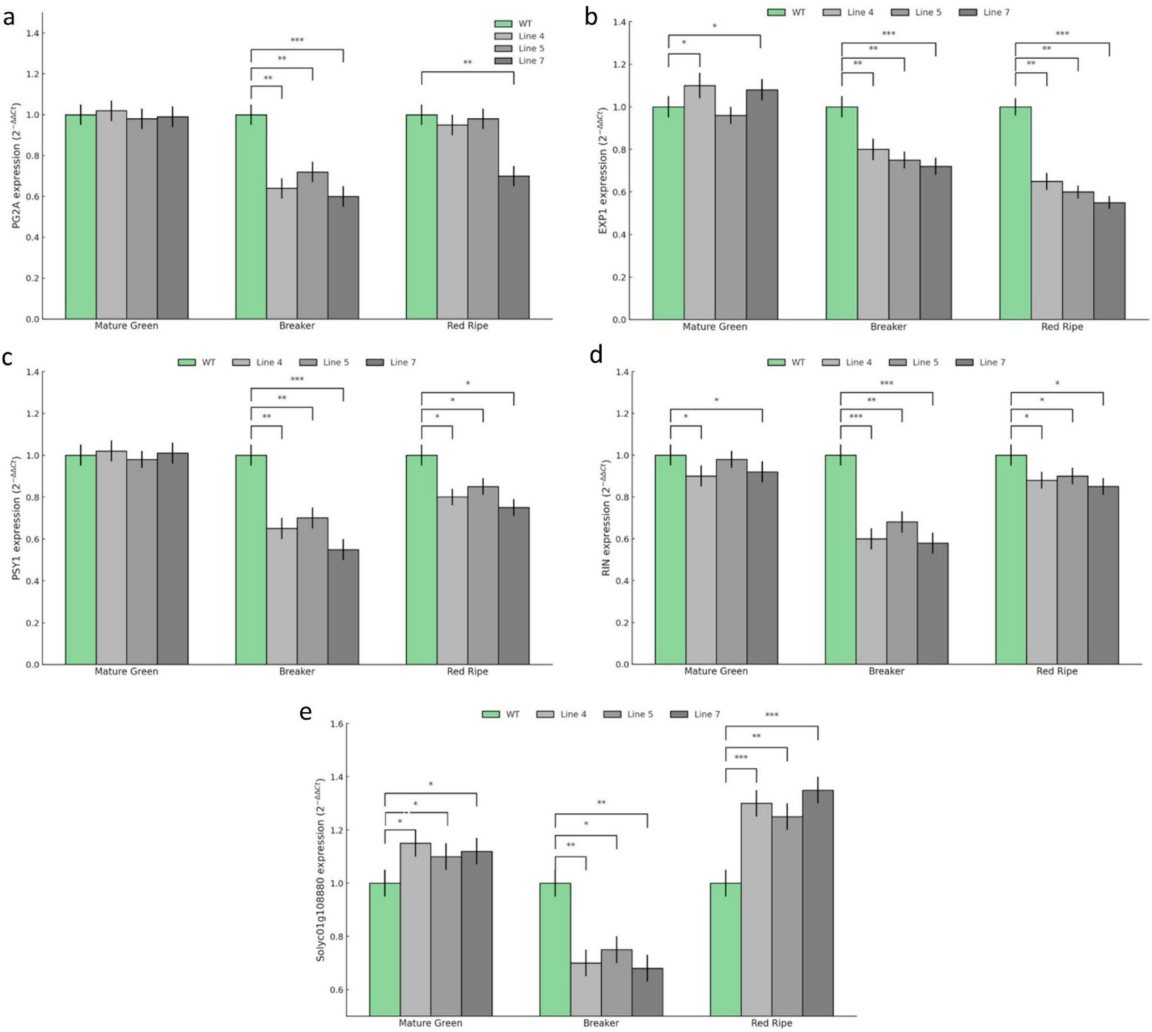
Expression analysis of ripening and cell wall-related genes in WT and *SlERF-RD1*-OE fruits. Relative mRNA abundance was determined by qRT-PCR for genes involved in cell wall disassembly (**a**, **b**), carotenoid biosynthesis (**c**), and the ripening regulatory hierarchy (**d**, **e**) across Mature Green (MG), Breaker (BR), and Red-Ripe (RR) stages. **a** *SlPG2A* (*Solyc03g111690*) **b** *SlEXP1* (Solyc06g051800) **c** *SlPSY1* (*Solyc03g031860*) **d** *SlRIN* (*Solyc05g012020*) **e** *Solyc01g108880* (Co-expressed target gene). Transcript levels were normalized to *SlActin* (*Solyc03g078400*) as an internal control. For each developmental stage, the expression level of WT was set to 1.0. Data represent the mean ± SD of three biological replicates. Different letters indicate statistically significant differences between WT and OE lines at each stage (*P < 0.05; **P < 0.01; ***P < 0.001; Student’s t-test)

To elucidate the molecular basis of the attenuated ripening and enhanced fruit firmness observed in *SlERF-RD1*-OE lines, we quantified the expression of key genes involved in cell wall disassembly, carotenoid biosynthesis, and the master regulatory hierarchy of tomato fruit. The enhanced firmness in OE lines was strongly corroborated by the significant down-regulation of major cell wall-modifying enzymes. While *SlPG2A* expression remained unchanged at the Mature Green (MG) stage, it exhibited a profound reduction at the Breaker (BR) stage, with levels decreasing to 0.58–0.69 fold across the three OE lines (Fig. 5a). This suppression persisted through the Red-Ripe (RR) stage, particularly in line #7 (0.72-fold), suggesting that *SlERF-RD1* primarily restricts pectin degradation during the onset of ripening. Similarly, *SlEXP1* displayed significant down-regulation at both the BR (0.63–0.78 fold) and RR stages (0.53–0.61 fold), indicating a sustained inhibition of cell wall loosening throughout the maturation process (Fig. 5b). The delayed pigmentation phenotype was molecularly confirmed by the attenuation of *SlPSY1* and the master regulator *SlRIN*. *SlPSY1* expression was significantly suppressed starting from the BR stage (0.55–0.64 fold) and remained consistently lower than the wild-type (WT) at the RR stage (0.74–0.83 fold) (Fig. 5c). Furthermore, *SlRIN* exhibited systemic down-regulation across all ripening stages, with the most substantial suppression occurring at the BR stage, where expression levels dropped to 0.59–0.68 fold relative to the WT (Fig. 5d). These findings suggest that *SlERF-RD1* delays ripening by acting upstream of the RIN-mediated regulatory circuit.

Interestingly, the co-expressed partner *Solyc01g108880* displayed a unique, biphasic expression pattern in response to *SlERF-RD1* overexpression. Following a modest induction at the MG stage (1.15–1.18 fold), a transient decrease was observed at the BR stage (0.64–0.73 fold). However, at the RR stage, its expression was robustly up-regulated across all OE lines, reaching 1.32-fold of WT levels (Fig. 5e). This late-stage induction, occurring alongside the suppression of *PG2A* and *EXP1*, suggests that *Solyc01g108880* may be associated with processes contributing to cell wall maintenance. However, this interpretation is based on expression patterns alone and requires further functional validation.

### In silico promoter and cis-acting element analysis of *SlERF-RD1* and its primary targets

**Fig. 6:**
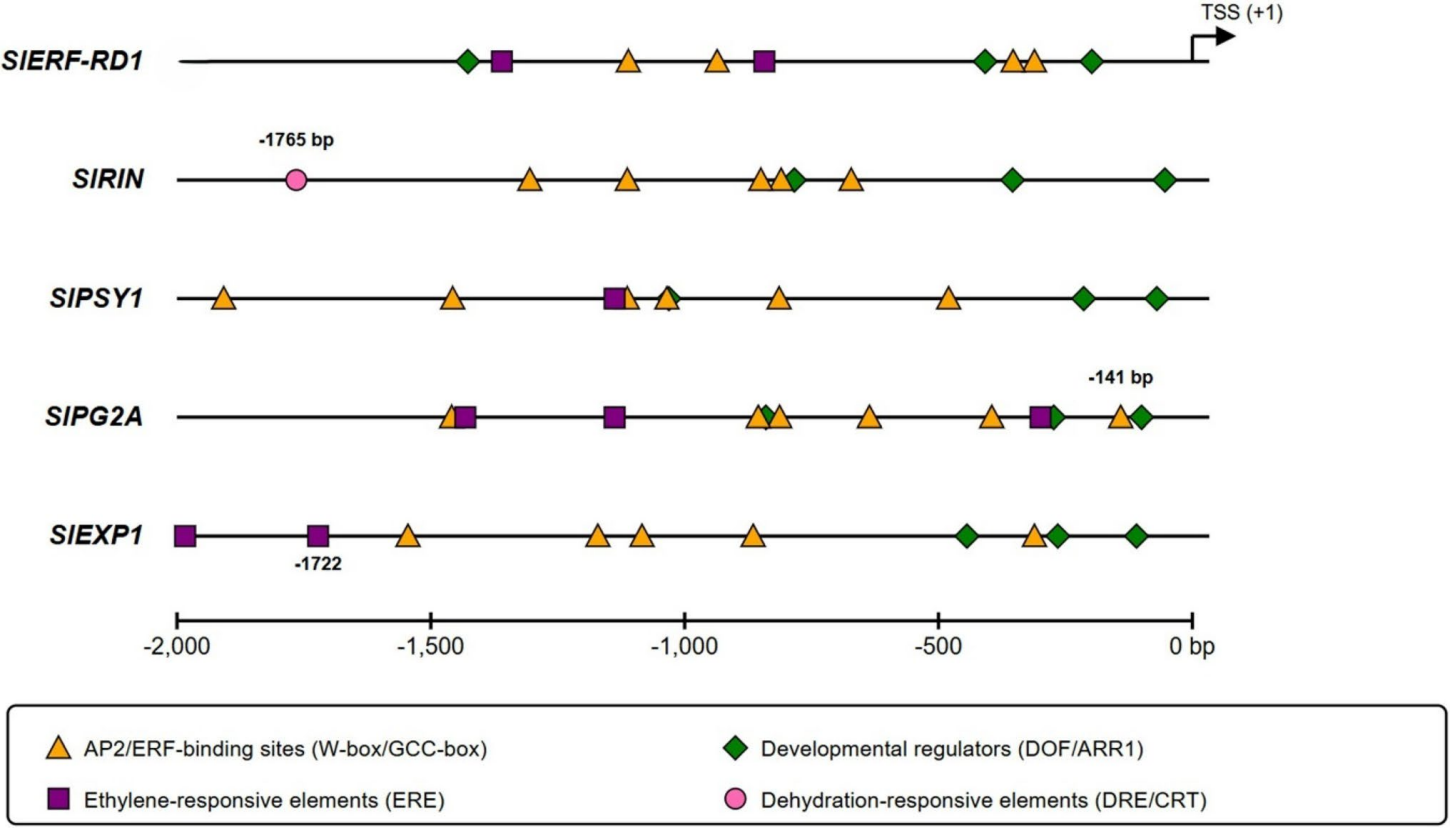
Comparative cis-acting element landscape of SlERF-RD1 and its primary regulatory targets. Schematic spatial distribution of regulatory motifs within the 2000 bp upstream regions of *SlERF-RD1, SlRIN, SlPSY1, SlPG2A,* and *SlEXP1* identified via the PLACE database. Colored markers indicate high-confidence binding sites: orange triangles represent AP2/ERF-binding sites (W-box/GCC-box), purple squares denote ethylene-responsive elements (ERE), and green diamonds mark developmental regulators (DOF/ARR1)

To explore the potential regulatory mechanism of *SlERF-RD1* over the ripening cascade, we performed a comprehensive cis-acting element profiling of the 2000 bp upstream regions of five key genes: the master regulator *SlRIN*, the carotenoid gene *SlPSY1*, the cell wall effectors *SlPG2A* and *SlEXP1*, and *SlERF-RD1* itself, using the PLACE database. Our analysis revealed a high density of canonical AP2/ERF binding motifs, specifically GCC-boxes (AGCCGCC) and W-boxes (TGACY/TTGAC), distributed across all five promoters (Fig. 6).

In the *SlRIN* (Solyc05g012020) promoter, we identified a critical drought-responsive element (DRE/CRT, RCCGAC) at position − 1765 bp and multiple W-box motifs (e.g., at − 671 and − 1112 bp). The presence of these high-affinity sites suggests that *SlERF-RD1* may directly bind to this master regulator to repress the core ripening hierarchy. Similarly, the *SlPSY1* (Solyc03g031860) promoter harbored a significant cluster of ERF-binding sites, including multiple WBOXNTERF3 motifs (e.g., at − 480, − 1,035, and − 1,908 bp), providing a molecular explanation for the altered carotenoid flux and delayed external pigmentation observed in overexpressing fruits.

Regarding fruit structural integrity, we detected a core GCC-box (AGCBOXNPGLB) in the *SlPG2A* (Solyc10g080210) promoter located only − 141 bp from the transcription start site (TSS). The close proximity of this motif to the TSS, combined with numerous W-boxes (e.g., at -813 and -855 bp), supports a transcriptional repression model that maintains firmness by limiting pectin degradation. Furthermore, in the *SlEXP1* (Solyc06g051800) promoter, the identification of multiple conserved W-box motifs and ethylene-responsive elements (ERELEE4, e.g., at − 1,722 and − 1,984 bp) provides a theoretical framework for the observed down-regulation of its transcript levels (up to 0.53-fold) in OE lines, highlighting how *SlERF-RD1* restricts cell wall loosening.

Additionally, the *SlERF-RD1* (Solyc02g077790) promoter itself contained multiple ethylene-responsive elements (ERELEE4) at − 843 and − 1,360 bp, alongside developmental motifs (DOFCORE and ARR1AT), consistent with its robust induction by ethylene and its pleiotropic role in coordinating plant architecture. Collectively, these in silico findings (Fig. 6) reinforce the role of *SlERF-RD1* as a strategic developmental coordinator that directly fine-tunes fruit ripening and softening by targeting key hierarchical and effector nodes.

## Discussion

The orchestration of tomato fruit ripening is a complex developmental process governed by a hierarchical network of transcription factors and the phytohormone ethylene (Giovannoni 2004). In this study, we identified and characterized *SlERF-RD1* (*Solyc02g077790*), a pivotal member of the AP2/ERF superfamily, which functions as a negative regulator of ripening while simultaneously enhancing fruit quality and structural integrity. Our characterization of *SlERF-RD1* further highlights the complexity of the ripening signaling network. The ethylene-mediated ripening cascade is not isolated but is deeply integrated with other diverse hormonal signals, such as cytokinin (Chen et al. 2025b) and melatonin (Xing et al. 2025). Within this extensive crosstalk, *SlERF-RD1* likely acts as a crucial hub, fine-tuning ethylene responses while preserving fruit structural integrity and plant architecture.

The identification of master regulators is a cornerstone in understanding the complex transcriptional landscape of tomato fruit ripening. In this study, our multi-layered bioinformatic pipeline (Fig. 1) prioritized *SlERF-RD1* (*Solyc02g077790*) as a core component of the ripening machinery. The integration of *SlERF-RD1* into ethylene-dependent signaling was evidenced by its high sensitivity to exogenous ethylene and its differential regulation in classic ripening mutants, such as *rin*, *nor*, and *Gr* (Fig. 1a). Such expression profiles are characteristic of key ripening-related transcription factors, including *SlERF.F12* (Deng et al. 2022) and *SlLBD33* (Hao et al. 2015), which act as transcriptional switches at the onset of maturation.

A pivotal finding in our in silico analysis was the high transcriptional synergy between *SlERF-RD1* and *Solyc01g108880* (Fig. 1c). The strong positive correlation (*R* = 0.91) in locular and placental tissues (Fig.S2) suggests that *SlERF-RD1* might orchestrate a specialized regulatory module during the transition to the red-ripe stage. In tomato, locular tissue and the placenta are the primary sites of ethylene biosynthesis initiation (Alba et al. 2005); therefore, the spatial enrichment of *SlERF-RD1* in these tissues strongly implies its involvement in the early signaling events that trigger the systemic ripening burst. Furthermore, the GO and KEGG enrichment results, which highlighted cell wall modification and secondary metabolism pathways, provided a functional roadmap that was later validated by our physiological and molecular findings in the overexpressing lines.

Overexpression of *SlERF-RD1* resulted in a distinctive morphological shift, characterized by a significantly more compact canopy and reduced plant height (Fig. 2). In tomato, plant architecture is tightly controlled by the balance between indeterminate and determinate growth signals, largely mediated by members of the AP2/ERF and MADS-box families (Shinozaki et al. 2018). The reduction in canopy diameter observed in our OE lines suggests that *SlERF-RD1* may interfere with internode elongation or lateral branching, a trait that is increasingly targeted in modern horticulture to enable high-density planting and improve light interception efficiency.

Remarkably, this vegetative compactness was coupled with an accelerated floral transition, evidenced by earlier anthesis in OE lines compared to WT (Fig. 3). This heterochronic development is further supported by the up-regulation of *SlSFT* (the florigen signal) and the suppression of *SlMC* (Fig. 3c). In tomato, the *SINGLE FLOWER TRUSS* (*SFT*) gene is the primary driver of floral induction, while *MACROCALYX* (*MC*) is involved in inflorescence architecture and sepal development (Molinero-Rosales et al. 2004; Xing et al. 2022). While classical MC mutations typically result in enlarged sepals, our OE lines unexpectedly exhibited an overall reduction in flower size. We propose that this reduced floral area is not a direct morphological consequence of MC suppression alone, but rather a developmental trade-off resulting from the prematurely activated SFT-MC axis. The highly accelerated floral meristem commitment likely restricts the temporal window required for adequate cell proliferation. Consequently, the antagonistic expression pattern of these core genes suggests that *SlERF-RD1* functions as a strategic developmental coordinator, reconfiguring the plant’s architecture to prioritize rapid reproductive success and early fruit set over prolonged vegetative expansion. Similar roles have been reported for other ERFs that integrate hormonal cues to rapidly time the switch from leaf production to flower formation.

Tomato fruit ripening is a climacteric process characterized by a massive burst of autocatalytic ethylene production (System II), which triggers downstream ripening effectors (Alba et al. 2005). Our physiological data revealed that overexpression of *SlERF-RD1* results in a significant reduction (40–47%) in ethylene emission and a substantial delay in fruit color transition (Fig. 4). This inhibitory effect is further corroborated by the striking down-regulation of *SlRIN* (*MADS-RIN*) and *SlPSY1* (Fig. 5c, d). *SlRIN* is a master transcription factor that occupies a central position in the ripening hierarchy, directly regulating genes involved in ethylene biosynthesis and carotenoid accumulation (Huang et al. 2022). The significant suppression of *SlRIN* transcript levels in *SlERF-RD1*-OE lines, particularly at the Breaker stage, suggests that *SlERF-RD1* acts as an upstream transcriptional repressor. By dampening the RIN-mediated regulatory node, *SlERF-RD1* effectively delays the onset of the ripening program. This is further reflected in the reduced expression of *SlPSY1*, the rate-limiting enzyme in the carotenoid pathway (Fray and Grierson 1993). The correlation between lower *PSY1* levels and the delayed pigmentation phenotype (Fig. 4a) confirms that *SlERF-RD1* modulates fruit color through the transcriptional control of the carotenoid biosynthetic flux. While SlPSY1 down-regulation suggests a reduced carotenoid biosynthetic flux, we cannot exclude the possibility of altered carotenoid partitioning in the absence of direct metabolite measurements. Therefore, these results should be interpreted as indicative of altered carotenoid metabolism rather than definitive changes in specific pigment composition. Similar to other negative regulators, such as *SlERF.F12* (Deng et al. 2022), *SlERF-RD1* appears to function as a fine-tuning mechanism that prevents the precocious activation of ripening-associated pathways. One of the most significant challenges in tomato breeding is decoupling the softening process from other ripening attributes to extend shelf-life without loss of flavor. Our results demonstrate that *SlERF-RD1* overexpression leads to superior fruit firmness (Fig. 4c), which is maintained even at the fully ripe stage. This phenotype is directly linked to the transcriptional suppression of two major cell wall-modifying enzymes: *SlPG2A* (*Polygalacturonase*) and *SlEXP1* (*Expansin*) (Fig. 5a, b). *SlPG2A* is responsible for pectin depolymerization in the middle lamella, a process traditionally viewed as a primary driver of tissue softening (Smith et al. 1988). Similarly, *SlEXP1* facilitates cell wall loosening by disrupting hydrogen bonds between cellulose microfibrils and hemicelluloses (Brummell et al. 1999). The significant down-regulation of these effectors in OE lines explains the structural integrity observed during post-harvest storage. A critical aspect of the *SlERF-RD1*-OE phenotype is its asynchronous maturation program. In several well-characterized ripening-deficient mutants, such as rin and nor, chronological progression (e.g., days post-breaker) is often associated with a substantial delay or partial arrest of ripening-related physiological processes, including ethylene production, pigment accumulation, and softening (Giovannoni 2004; Klee and Giovannoni 2011; Pech et al. 2012). In contrast, *SlERF-RD1* overexpression appears to decouple specific ripening modules. While external pigmentation and cell wall disassembly are significantly repressed, internal metabolic maturation, evidenced by the successful accumulation of internal lycopene, soluble solids (°Brix), and ascorbic acid, proceeds robustly. This targeted uncoupling results in a distinct "firm-ripe" phenotype rather than a uniform developmental delay. Consequently, *SlERF-RD1* offers a unique genetic tool to dissect the regulatory branches of tomato ripening, successfully prioritizing post-harvest structural integrity and nutritional density without compromising primary metabolic readiness.

More interestingly, our study identified *Solyc01g108880* as a strongly co-expressed candidate gene exhibiting a distinct biphasic expression pattern during fruit ripening (Fig. 5e). Following a moderate induction at the MG stage, its expression decreased at the BR stage and was subsequently up-regulated at the RR stage in *SlERF-RD1-OE* lines. While this expression pattern suggests a potential involvement in late-stage ripening processes, it is important to emphasize that co-expression alone does not establish a direct functional or regulatory relationship. Therefore, the association between *SlERF-RD1* and *Solyc01g108880* should be interpreted as a hypothesis-generating observation rather than definitive evidence of regulatory interaction. Future studies employing functional approaches, such as loss- or gain-of-function analyses, will be necessary to determine whether Solyc01g108880 plays a direct role in cell wall dynamics or other ripening-associated processes. Notably, such co-expression-based candidate identification has been widely used as an initial step in defining regulatory modules in plant transcriptomic studies, although it requires subsequent experimental validation.

Beyond structural integrity, the overexpression of *SlERF-RD1* positively influenced the internal quality parameters of the fruit. Our data showed a significant increase in SSC/°Brix and Vitamin C levels at the Red-Ripe stage in OE lines (Fig.S3). In many tomato mutants where ripening is delayed, there is often a negative correlation with flavor and nutrient accumulation (Beckles 2012). However, *SlERF-RD1* appears to bypass this quality trade-off. The identification of putative transcriptional targets is a key step toward understanding the regulatory networks that govern fruit maturation. In this study, our in silico analysis suggests that *SlERF-RD1* may function as a direct molecular repressor by targeting the promoters of several key ripening- and softening-related genes. The presence of a high-confidence DRE/CRT element at − 1765 bp within the SlRIN promoter indicates the potential for *SlERF-RD1* to modulate the ripening hierarchy at an upstream level. This possible interaction could provide a molecular basis for the systemic attenuation of the ripening program observed in our overexpression lines, potentially mirroring the repressive roles reported for other ERF family members on master regulators. The observed maintenance of fruit firmness in *SlERF-RD1*-OE lines might be further explained by the promoter architectures of SlPG2A and SlEXP1. We identified a core GCC-box in the SlPG2A promoter, located remarkably close (-141 bp) to the transcription start site, alongside multiple W-box motifs within the SlEXP1 promoter. The proximity and density of these AP2/ERF-specific binding sites suggest that *SlERF-RD1* could potentially restrict pectin degradation and cell wall loosening through direct transcriptional suppression. Furthermore, the identification of multiple ethylene-responsive elements (ERELEE4) in the *SlERF-RD1* promoter might account for its rapid induction by ethylene, possibly forming a negative feedback loop that modulates the pace of fruit softening. Collectively, these findings support a model where *SlERF-RD1* may act as a strategic developmental coordinator, potentially integrating hormonal cues to fine-tune the balance between fruit structural integrity and maturation.

The atypical orange/pale external phenotype of the *SlERF-RD1*-OE fruits raises intriguing questions regarding pigment biosynthesis. While the macroscopic exterior appears delayed, cross-sections reveal that red pigment accumulation successfully proceeds within the internal tissues. Therefore, the external orange appearance is likely not driven by a global shift in carotenoid flux, but rather by an asynchronous ripening delay restricted to the outer pericarp and epidermis. This tissue-specific delay is accompanied by an apparent reduction in epidermal yellow flavonoids, leading to the atypical visual color. Future tissue-specific metabolic profiling will be necessary to fully map the biochemical nature of this superficial pigmentation delay. The higher °Brix levels suggest that the delay in the climacteric burst allows for a more prolonged period of carbohydrate translocation and accumulation within the fruit sink. Furthermore, the elevated Vitamin C levels suggest that *SlERF-RD1* may modulate the antioxidant machinery, potentially in response to altered ethylene signaling, similar to the metabolic shifts observed in high-quality tomato genotypes (Stevens et al. 2007).

In conclusion, we propose a regulatory model where *SlERF-RD1* serves as a developmental coordinator. By acting as a transcriptional repressor of master regulators, such as *SlRIN*, and cell wall effectors, including *SlPG2A* and *SlEXP1*, it delays the rapid phase of fruit softening and pigmentation. While the overexpression of *SlERF-RD1* successfully enhances fruit firmness and primary nutritional density (elevated °Brix and ascorbic acid), we acknowledge significant pleiotropic trade-offs that currently limit its direct commercial application. Most notably, the accelerated floral transition and premature meristem commitment restrict cellular proliferation, resulting not only in smaller flowers but also in correspondingly smaller mature fruits. This reduction in overall fruit size and yield represents a critical commercial bottleneck. Furthermore, while primary metabolites are improved, the impact of *SlERF-RD1* on the biosynthesis of complex flavor volatiles remains unknown and necessitates future comprehensive metabolomic profiling. Therefore, rather than proposing *SlERF-RD1* overexpression as an immediate agronomic solution, we propose that this transcription factor serves as a crucial molecular hub. Future precision breeding strategies, such as CRISPR/Cas9-mediated promoter editing, could be deployed to delicately fine-tune its expression levels, aiming to uncouple the highly desirable delayed-softening traits from the negative developmental penalties on fruit size. It is important to consider that the functional characterization of *SlERF-RD1* in this study was conducted using the cv. Crocker genetic background. While this cultivar is well-suited for stable transformation and controlled phenotypic analysis, transcriptional regulation and developmental responses may vary across different tomato genotypes. Given that the in silico analyses were based on publicly available datasets derived from multiple genetic backgrounds, potential genotype-specific effects cannot be fully excluded. Therefore, the regulatory role of *SlERF-RD1* described here should be interpreted within the context of the Crocker background, and further validation across diverse cultivars will be necessary to assess its broader applicability. While overexpression analysis provides important insights into the regulatory potential of *SlERF-RD1* as a negative modulator of ripening, it does not fully resolve its endogenous functional role. Loss-of-function approaches, such as CRISPR/Cas9-mediated knock-out or knock-down strategies, would be essential to validate the direct contribution of *SlERF-RD1* to ripening control and plant developmental processes. In the present study, overexpression was prioritized to evaluate the potential of *SlERF-RD1* as a regulatory “brake” within the ripening network. Future studies integrating complementary loss-of-function approaches will be necessary to fully elucidate its biological function and to confirm the causative relationships suggested by our findings.

## Supplementary Information

Below is the link to the electronic supplementary material.

Supplementary file1 (DOCX 1598 KB)

Supplementary file2 (XLSX 13 KB)

## Data Availability

All data supporting the findings of this study are included within the article and its supplementary materials. Sequence data for the *SlERF-RD1* gene can be found in the Sol Genomics Network (SGN) database under the accession number *Solyc02g077790*. Raw transcriptomic data used for in silico analyses are available in the public repositories referenced in the text.

## Declarations

### Generative AI and AI-assisted technologies

During the preparation of this manuscript, the authors utilized artificial intelligence (AI) tools specifically for language editing and structural refinement to ensure academic clarity and grammatical precision. The authors reviewed and edited the content as needed and took full responsibility for the final scientific accuracy and integrity of the work presented.
