# Supplementary material for: SlERF-RD1 acts as a developmental coordinator integrating plant canopy architecture and the ethylene-mediated ripening cascade in tomato
Source: Plant Cell Rep. 2026 May 17;45(6):162. doi: 10.1007/s00299-026-03848-z (PMC13180767; doi:10.1007/s00299-026-03848-z)
Supplement: Supplementary file 1 — Supplementary file1 (DOCX 1598 KB) [file 299_2026_3848_MOESM1_ESM.docx]

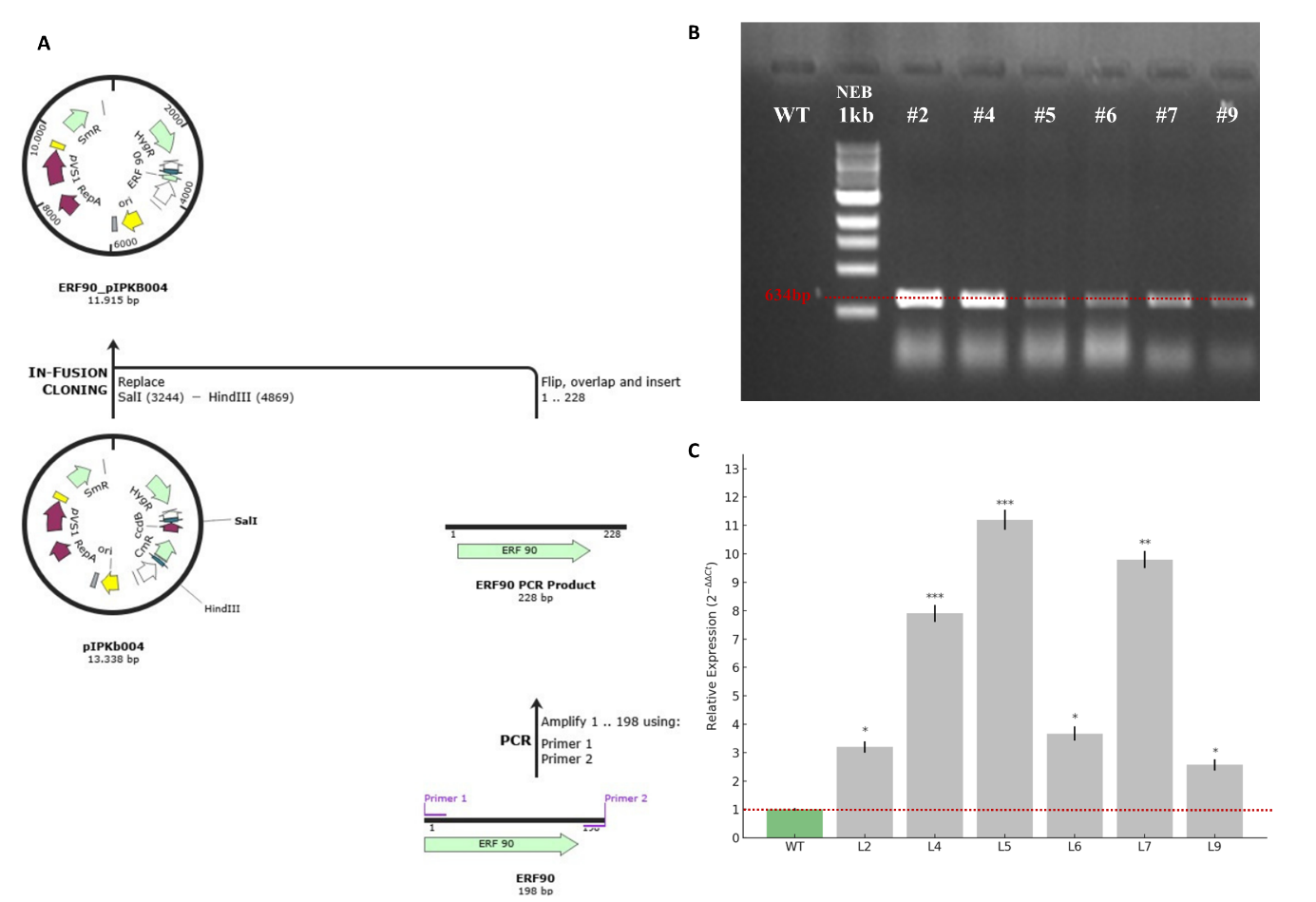


**Fig S1. Generation and molecular confirmation of *SlERF-RD1*-overexpressing tomato lines.** Generation and molecular confirmation of *SlERF-RD1*-overexpressing tomato lines. (A) Schematic representation of the *SlERF-RD1*_pIKB004 expression cassette. (B) Genomic PCR screening of putative transgenic lines; the ~634 bp band indicates successful T-DNA integration. (C) Relative transcript abundance of *SlERF-RD1* in WT and selected OE lines measured by qRT-PCR. Data represent mean ± SEM of three biological replicates. Asterisks indicate significant differences relative to WT based on one-way ANOVA followed by Tukey’s post-hoc test (* P < 0.05; ** P < 0.01; *** P < 0.001).


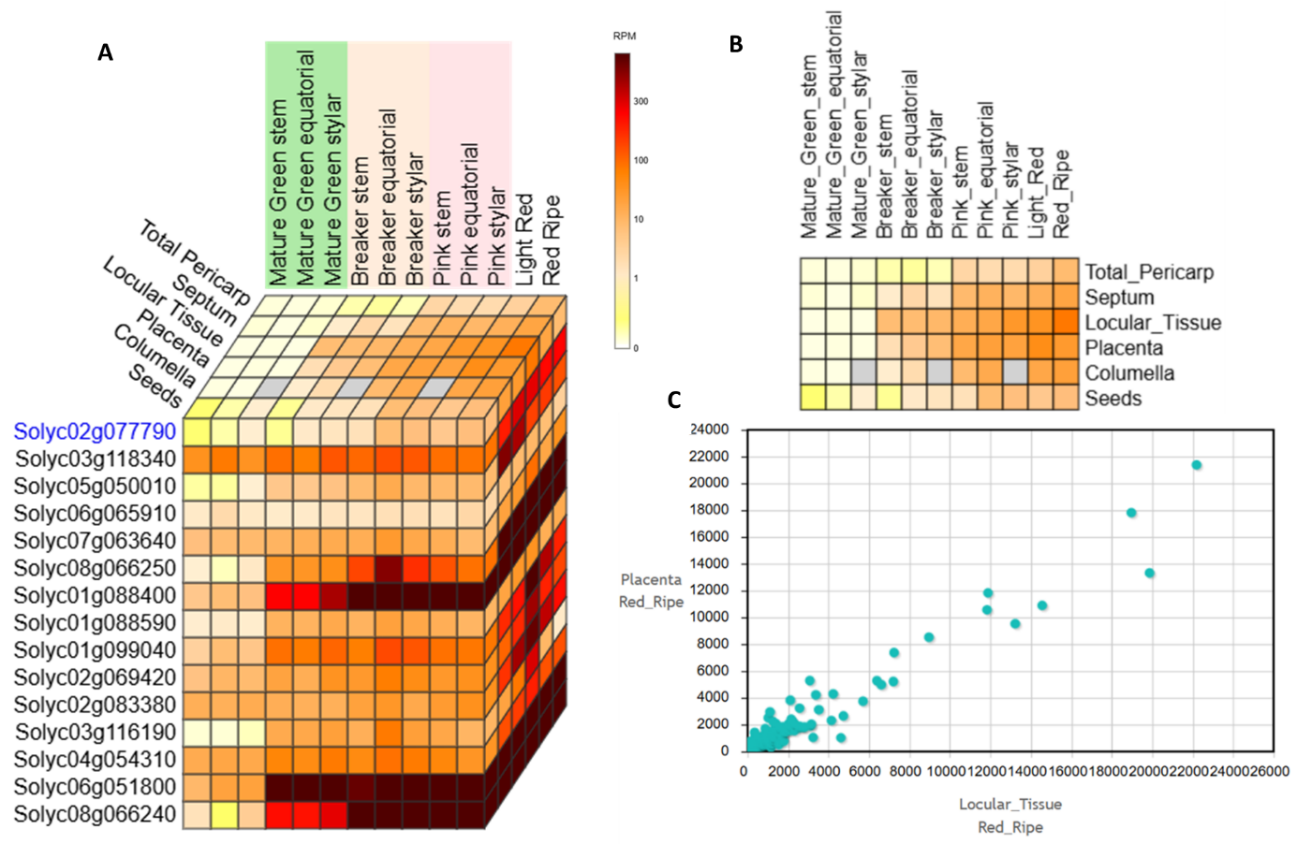
**Figure Fig S2**. **Spatial and tissue-specific expression landscape of SlERF-RD1.** (A-B) Heatmap analysis integrating transcript abundance across distinct fruit tissues (pericarp, septum, locular tissue, etc.) and developmental stages. (C) Regression analysis showcasing a strong positive correlation (R = 0.91) between *SlERF-RD1* expression in locular and placental tissues at the red-ripe stage, suggesting synchronized regulatory roles. Statistical significance for correlation was determined using Pearson’s product-moment correlation coefficient.


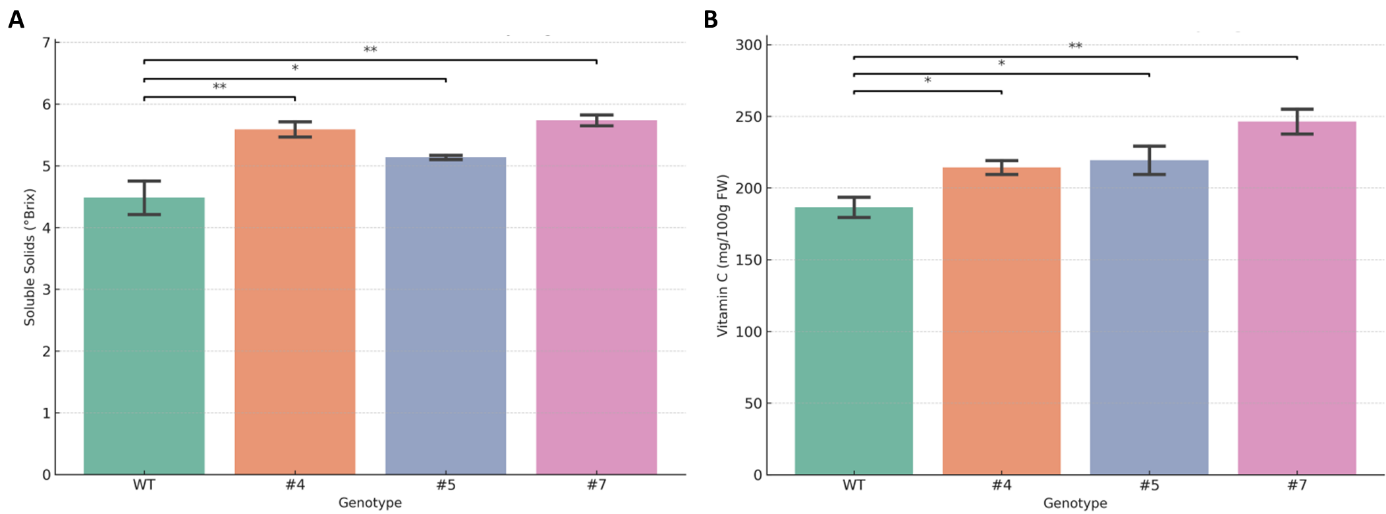


**Fig S3. Impact of SlERF-RD1 on soluble solids and antioxidant content.** (A) Soluble solids content (°Brix) and (B) Vitamin C (ascorbic acid) concentration (mg/100g FW) in WT and OE red-ripe fruits. Data represent mean ± SE (n = 3 biological replicates). Different letters indicate significant differences (P < 0.05, Tukey’s HSD).


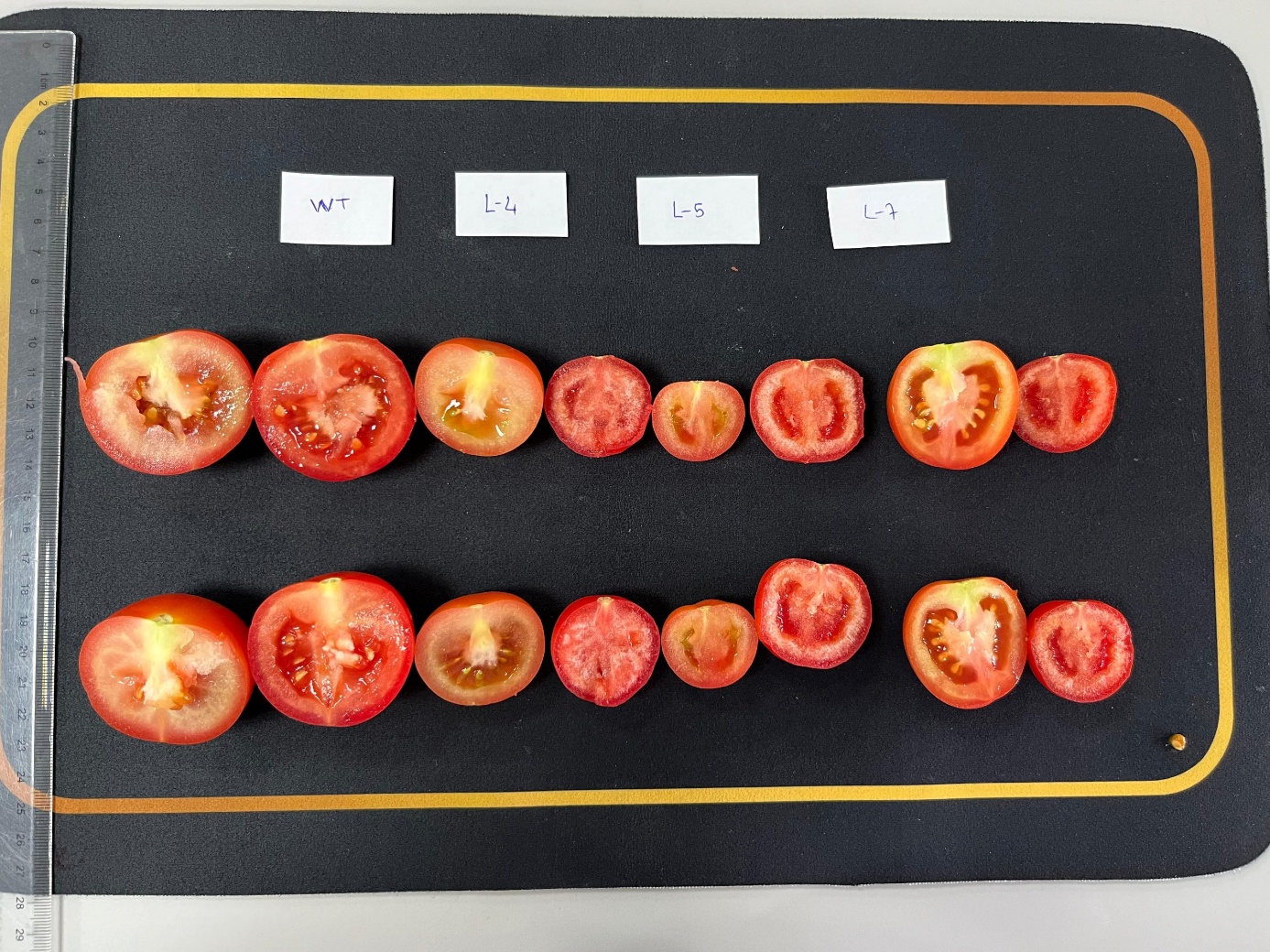


**Fig. S4. Asynchronous spatial ripening and "inside-out" pigmentation delay in *SlERF-RD1*-OE fruits.** Representative cross-sections of wild-type (WT) and transgenic lines (OE-4, OE-5 and OE-7) at the Red-Ripe (RR) stage (7 days post-breaker). While WT fruits exhibit uniform red pigmentation across all fruit layers, the OE fruits display a distinct spatial decoupling of pigment accumulation. The internal tissues (columella, locular gel, and inner pericarp) successfully ripen and accumulate red pigments (lycopene) comparably to the WT.
